# Balloon Deflation Strategy during Primary Percutaneous Coronary Intervention in Acute ST-Segment Elevation Myocardial Infarction: A Randomized Controlled Clinical Trial and Numerical Simulation-Based Analysis

**DOI:** 10.1155/2020/4826073

**Published:** 2020-09-07

**Authors:** Jun Gu, Yang Zhuo, Tian-jiao Liu, Jie Li, Zhao-fang Yin, Zuo-jun Xu, Li Fan, Qing He, Kan Chen, Hua-su Zeng, Xiao-fei Wang, Yu-qi Fan, Jun-feng Zhang, Fu-you Liang, Chang-qian Wang

**Affiliations:** ^1^Department of Cardiology, Shanghai Ninth People's Hospital, Shanghai Jiaotong University School of Medicine, Shanghai, China; ^2^School of Naval Architecture, Ocean and Civil Engineering, Shanghai Jiao Tong University, Shanghai, China; ^3^Institute for Personalized Medicine, Sechenov University, Moscow, Russia

## Abstract

**Background:**

Primary percutaneous coronary intervention (PCI) is the best available reperfusion strategy in patients with acute ST-segment elevation myocardial infarction (STEMI). However, PCI is associated with a serious problem known as no-reflow phenomenon, resulting in poor clinical and functional outcomes. This study aimed to compare the influences of different balloon deflation velocity on coronary flow and cardiovascular events during primary PCI in STEM as well as transient hemodynamic changes in in vitro experiments. *Method and Results*. 211 STEMI patients were randomly assigned to either a rapid or a slow balloon deflation group during stent deployment. The primary end point was coronary flow at the end of PCI procedure, and secondary end points included myocardial infarct size. Transient hemodynamic changes were evaluated through an in vitro experimental apparatus and a computer model. In clinical practice, the level of corrected TIMI frame count (cTFC) in slow balloon deflation after primary PCI was significantly lower than that of rapid balloon deflation, which was associated with smaller infarct size. Numerical simulations revealed that the rapid deflation led to a sharp acceleration of flow in the balloon-vessel gap and a concomitant abnormal rise in wall shear stress (WSS).

**Conclusion:**

This randomized study demonstrated that the slow balloon deflation during stent implantation improved coronary flow and reduced infarct size in reperfused STEMI. The change of flow in the balloon-vessel gap and WSS resulted from different balloon deflation velocity might be partly accounted for this results.

## 1. Introduction

Although timely and successful reperfusion with primary percutaneous coronary intervention (PCI) is the most effective method for reducing infarct size and improving the outcome in patients with acute ST-segment elevation myocardial infarction (STEMI) [1, 2], PCI is also associated with a serious problem known as no-reflow phenomenon, which significantly attenuates the beneficial *effects* of reperfusion therapy, resulting in poor clinical and functional outcomes. This phenomenon may develop in 5–50% of STEMI patients during primary PCI [3–6]. *No-reflow is thought to be caused by a variety of pathophysiological factors, such as distal embolization, ischemia-reperfusion injury, and the susceptibility of coronary microcirculation to injury* [3–6].

Optimal stent deployment is an important component in determining outcomes with primary PCI for STEMI. Currently, rapid balloon deflation (from higher inflation pressure abruptly to negative pressure) during coronary stent deployment is universally performed. However, the rapid stent balloon deflation might lead to more significant siphonic effects and rapid changes in coronary hemodynamics, which was probably associated with distal embolization and microcirculation dysfunction [7]. And *the embolization of plaque debris* may be an important cause of the no-reflow or slow-flow phenomenon [8]. Until now, there is a paucity of data *to evaluate* the association of balloon deflation strategies during stent deployment with coronary flow and clinical outcomes in patients presenting with STEMI treated with primary PCI. The primary finding of this study is that the use of slow deflation strategy led to favorable coronary flow and infarct size compared with conventional rapid deflation for stent deployment. Additionally, an in vitro experimental apparatus combined with a computer model indicated that the change of flow velocity in the balloon-vessel gap and wall shear stress (WSS) resulted from different balloon deflation strategy might be partly accounted for this clinical benefit.

## 2. Methods

### 2.1. Clinical Study Design and Patients

This was a single-center, prospective, randomized, and controlled study. After the patients had given informed consent, they were randomly allocated to *either the rapid or slow balloon deflation group* through pre-established sealed envelopes in a 1 : 1 ratio after diagnostic angiography. Imaging investigators, *statisticians*, and also patients were blinded to the allocated group. Numbered sealed envelopes that contained the study group assignment were distributed to each catheterization laboratory and were opened after informed consent had been obtained. The authors designed the study, and the local institutional review board approved the trial protocol. Inclusion criteria were as follows: (1) patients ≥18 and ≤80 years of age; (2) STEMI patients presenting within 12 h after chest pain onset, with ST-segment elevation ≥0.1 mV in two contiguous leads on 12-lead electrocardiogram or new left bundle branch block; (3) target lesion in a native coronary vessel with a reference diameter of 2.5 to 4.0 mm; (4) visual residual diameter stenosis ≥70% before stent implantation; (5) voluntary participation and signed informed consent. Exclusion criteria were (1) cardiogenic shock; (2) acute occlusive lesion of left main, bypass grafting, and in-stent; (3) high risk of bleeding or allergy to aspirin, heparin, clopidogrel, ticagrelor, or rapamycin; (4) complicated with other serious diseases (malignant tumor, organ transplantation, or candidate); (5) severe liver and kidney dysfunction; (6) noncardiac comorbid conditions with a life expectancy <1 year or that may result in protocol noncompliance (per site investigator's medical judgment).

PCI was performed according to standard techniques. Before the index procedure, all patients received 300 mg aspirin and 600 mg clopidogrel or 180 mg ticagrelor as loading doses. Unfractionated heparin was administered intravenously before PCI, and the active clotting time was maintained at >250 seconds throughout the procedure. Thrombus aspiration, *predilation* before stenting or *postdilation* after stenting, or use of glycoprotein IIb/IIIa inhibitors was left to the operators' discretion. In the rapid deflation group, the stent balloon was deflated to negative pressure abruptly during stent deployment. In the slow deflation group, the stent balloon was deflated to zero atmospheres and then negative pressure slowly (2 atmospheres per second). This balloon deflation strategy was also applied in stent *postdilation*, if necessary. After reperfusion, PCI was completed according to the physician's judgment with respect to patient status.

### 2.2. Corrected TIMI Frame Count (cTFC)

Diagnostic coronary angiography and PCI procedure were performed by the insertion of a 6-French arterial sheath via the radial artery using the Seldinger method. Angiography CDs of the patients were reviewed by two interventional cardiologists who were blinded to all data other than the coronary angiograms. *TIMI frame count (TFC) was determined with a digital system in the catheterization laboratory. TFC refers to the numbers of cine-frames required for contrast to reach a standardized distal coronary landmark in the culprit vessel* [9]. *cTFC means that the TFC of left anterior descending (LAD) must be corrected by dividing it into 1.7 because of the longer length of LAD*. The cTFC count is considered as 100 frames for an occluded vessel [10]. Enrolled patients received automated contrast injection with the ACIST device (ACIST Medical Systems Inc., Eden Prairie, MN) for cTFC evaluation.

After the procedure, creatine kinase (CK)/CK-MB and troponin I (TNI) *were measured* before PCI and every 6 hours for 24 hours after the index procedure, thereafter, CK-MB and TNI were measured once daily to document the peak value. Besides, the area under the curve (AUC) of the release of CK-MB was approximated as a surrogate marker of infarct size. GraphPad Prism 5.02 was used to measure the AUC. All patients were recommended to receive optimal pharmacological therapy, including statins, *β*-blockers, or renin-angiotensin system blockade, following the current guidelines. Dual antiplatelet therapy (aspirin 100 mg/d plus clopidogrel 75 mg/d or ticagrelor 90 mg/bid) was recommended for at least 12 months.

### 2.3. Study End Points

The primary end point was coronary flow (determined by cTFC) after the procedure. Secondary end points included myocardial infarct size and major adverse cardiac events (MACEs, a composite of death, myocardial infarction, revascularization, heart failure, and rehospitalization) at 30 days. No-reflow was defined as the culprit coronary artery flow less than TIMI flow grade 2 during or at the end of the PCI as revealed by coronary angiogram.

### 2.4. Hemodynamic Characteristics upon Different Balloon Deflation Strategy

**Figure 1:**
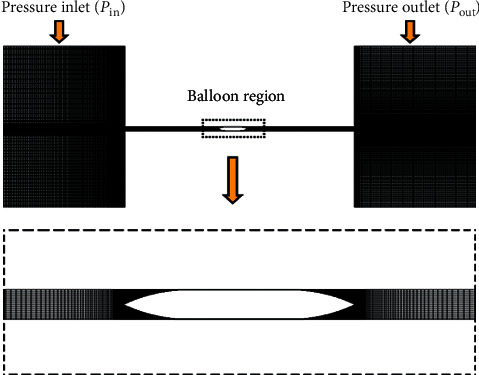
Computational mesh model.

*On the basis of our previous hemodynamic study* [11, 12], we innovatively established a hemodynamic in vitro study model of coronary balloon deflation [13]. An in vitro experimental apparatus was built, in which a high-speed camera was used to take snapshots of balloon deformation and flow field (dyed water) during balloon deflation. Subsequently, image processing techniques were employed to derive the parameters of balloon deformation and estimate the flow velocity downstream from the balloon (Figure 1). A computer model of the experimental apparatus was constructed, with the incorporation of the measured balloon deformation data, simulated the balloon deflation process under various perfusion pressure and fluid conditions. The basic reference velocity of balloon deformation (vb) was measured by in vitro balloon deformation experiment (*P*_in_ = 120 mmHg and *P*_out_ = 20 mmHg) [13–15]. In the numerical calculation, vb was reduced by 50% and increased by 100% respectively to simulate the different conditions of balloon deflation.

### 2.5. Statistical Analysis

To test the hypothesis that slow stent balloon deflation adjunctive to primary PCI is superior to conventional rapid balloon deflation for improving coronary reperfusion, we assumed that slow deflation would decrease the cTFC by 4 based on our previous work. On the basis of the estimated improvement in the primary end point, we selected a target sample size of at least 192 subjects, which would provide 80.0% power at the 0.05 significance level to detect anticipated differences and to offer some protection for coronary flow. Statistical analysis was performed using SPSS Statistical Software, version 22.0 (SPSS Inc., Chicago, IL, USA). Arithmetic means ± standard deviations were calculated for quantitative variables, while qualitative variables were given as frequency and percentage (%). For quantitative variable analysis, the *t*-test was used. A two-sided chi-square test was used to compare qualitative variables. Differences in clinical endpoints between rapid and slow balloon deflation were tested with the *t*-test or chi-squared test. All values were two-tailed, and a *P* value <0.05 was considered statistically significant.

## 3. Results

### 3.1. Study Population

**Figure 2:**
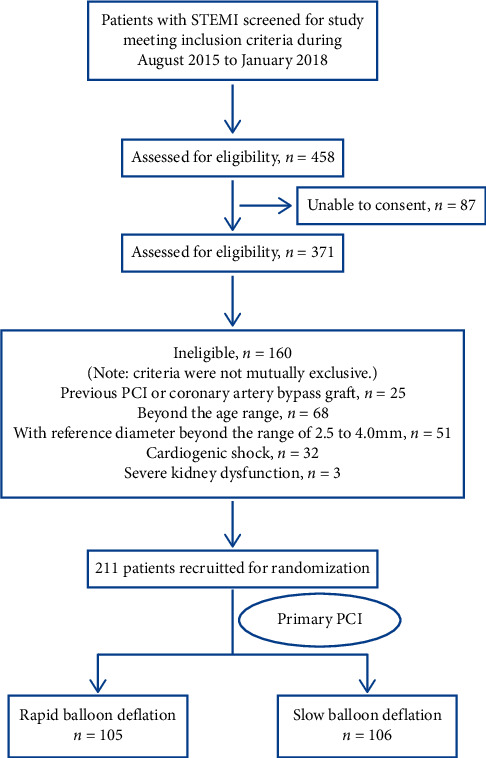
Flowchart of the clinical study protocol.

Between August 2015 and January 2018, 458 patients were considered to be eligible for the present study. Of these, 247 patients were not included for the following reasons: previous PCI or coronary artery bypass graft (CABG) (*n* = 25), beyond the age range (*n* = 68), with reference diameter beyond the range of 2.5 to 4.0 mm (*n* = 51), cardiogenic shock (*n* = 32), severe kidney dysfunction (*n* = 3), or involuntary participation (*n* = 87) (Figure 2). Data *were* thus presented for 211 patients (105 in the rapid balloon deflation group, and 106 in the slow balloon deflation group). There was no difference between the two groups with respect to the baseline population characteristics (Table 1). Duration of ischemia, coronary angiography features, and treatments administered either before and during PCI or at discharge were comparable between groups (Tables 2 and 3).

### 3.2. Coronary Flow and Infarct Size

For the primary endpoint, final cTFC after primary PCI in the slow balloon deflation group decreased significantly compared to the rapid balloon deflation group (24 ± 7 vs 27 ± 9, *P* = 0.015). In addition, the change of cTFC from baseline was much more prominent in the slow deflation (65.9 ± 16.5 vs 61.0 ± 18.2, *P* = 0.045). And considerable improvements of coronary flow (cTFC) were observed in the slow deflation compared with the rapid deflation immediately after stent deployment (25 ± 8 vs 28 ± 9, *P* = 0.035). Although there was less “no-reflow” *in the slow deflation relative to the rapid deflation*, no significant difference was found (3/106 vs 8/105, *P* = 0.118). Finally, the peak for serum CK/CK-MB or TNI release was significantly lower *in the slow deflation versus that in the rapid deflation*(Table 4). *And the AUC of CK-MB (0–72 h) in the slow deflation was significantly less than that in the rapid deflation (8302.2 ± 3916.5 vs 9570.7 ± 5122.3,P = 0.022)*.

### 3.3. Left Ventricular Ejection Fraction and MACEs

**Figure 3:**
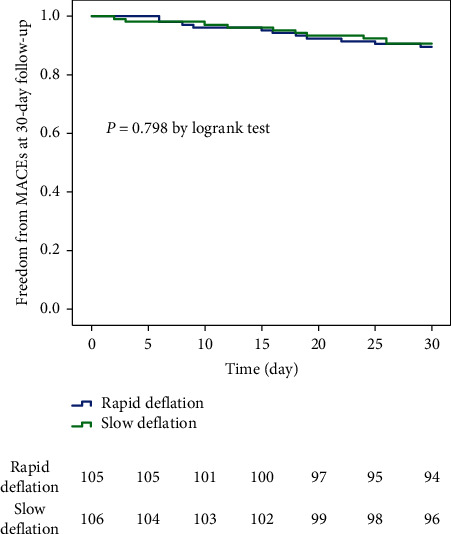
Kaplan–Meier curves of freedom from the occurrence of major adverse cardiovascular events (MACEs) for different balloon deflation strategy during 30-day follow-up.

Echocardiography was performed at 7 days after reperfusion. *Regarding the left ventricular ejection fraction (LVEF), there was no obvious difference between the two groups (slow vs rapid: 51.9 + 8.4% vs 50.1 + 7.9%,P = 0.089). Compared to the rapid balloon deflation, the slow balloon deflation did not lead to lower rates of 30-day MACEs events*(Table 5 and Figure 3).

### 3.4. Hemodynamic Characteristics by Numerical Simulations

**Figure 4:**
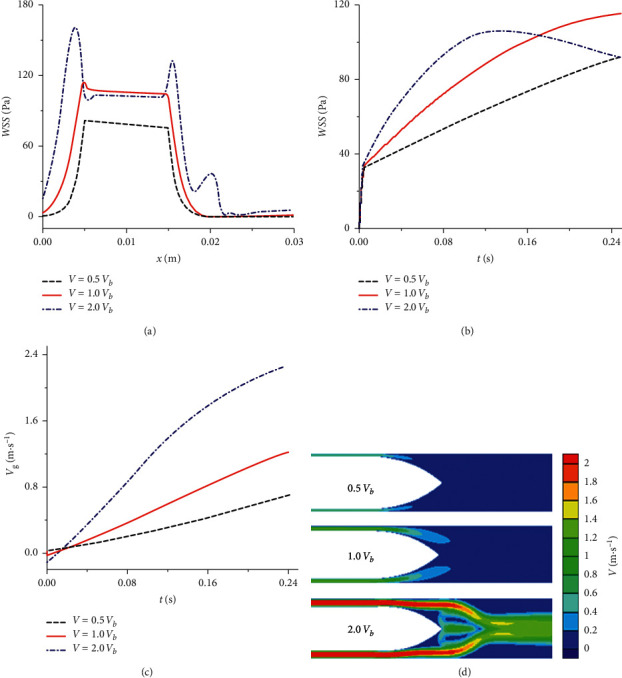
Comparisons of simulated flow velocities and vascular wall shear stress (WSS) under various balloon deflation velocities. (a) Simulated vascular WSS with the fluid and perfusion pressure along the balloon; (b) simulated vascular WSS in the balloon area under different velocity of balloon deflation; (c) simulated flow velocity in the balloon-vessel gap under different velocity of balloon deflation; (d) simulated flow velocity contour maps under different velocity of balloon deflation.

Numerical simulations revealed that under the condition of vb, about 0.18 s after the balloon deflation, the WSS downstream of the balloon reached 1.75 Pa, close to the physiological value of blood flow velocity in the coronary artery [15]; however, the WSS (110–115 Pa) in the area of balloon reached over 60 times of their physiological values (Figure 4(a)). Moreover, rapid balloon deflation led to a sharp acceleration of flow in the balloon-vessel gap (Figures 4(c) and 4(d)) and a concomitant abnormal rise in WSS (Figure 4(b)).

## 4. Discussion

In this prospective, randomized, and controlled trial, the slow balloon deflation during stent deployment with primary PCI improved coronary flow and decreased infarct size to a greater extent than conventional rapid deflation strategy. However, the rate of 30-day MACEs was not significantly different between the two groups. The favorable effect of slow balloon deflation might be associated with its influence on hemodynamic characteristics.

STEMI remains one of the leading causes of death worldwide. The main goal in the treatment of STEMI is to recanalize the culprit artery occlusion at an early stage. Primary PCI has been shown to be the most effective reperfusion strategy in the treatment of STEMI. When no-reflow occurs during primary PCI, it significantly attenuates the beneficial impact of reperfusion therapy, resulting in poor clinical and functional outcomes. Previous studies indicate that no-reflow is a multifactorial phenomenon, and its mechanisms include pre-existing microvascular dysfunction, distal micro-thrombo-embolization, ischemic injury, reperfusion injury, and individual susceptibility [3–6].

The restoration of coronary blood flow in STEMI reperfusion can paradoxically induce additional myocardial damage. The characteristics of clinically identified features of this reperfusion injury might be reversible and transient, such as arrhythmias or myocardial stunning, or irreversible, such as myocardial infarction or microvascular obstruction [16]. The myocardial damage during PCI might be associated with balloon deflation strategy during stent deployment. During stent implantation, the rapid deflation of the stent balloon might lead to increased coronary blood flow volatility and local shear stress, which was probably related with ischemia-reperfusion injury [16]. Our in vitro experiment and computer model indicated that WSS in the balloon area reached over 60 times of their physiological values during balloon deflation. More importantly, compared with the slow balloon deflation, the rapid deflation led to a sharp acceleration of flow in the balloon-vessel gap and a concomitant abnormal rise in WSS. In theory, the rapid balloon deflation might increase the risk of falling off of plaque fragments and microcirculation embolization in the downstream of coronary artery.

Previous studies have reported that high WSS is closely related to more vulnerable plaque [17]. High WSS assessed by intravascular ultrasound images is also associated with longitudinal development of high-risk plaque, including intraplaque necrotic core or expansive remodeling [15, 18, 19]. Indeed, higher WSS in the proximal segments of atherosclerotic lesions is predictive of myocardial infarction in patients with stable coronary artery disease (CAD) [18, 20]. *During primary PCI for STEMI, the rapid balloon deflation leads to higher WSS, which might be associated with adverse cardiovascular events*. Moreover, fluctuations in coronary flow during reperfusion result in flow disturbances, in parallel, endothelial proinflammatory activation and vascular leakage occur [21, 22]. One study indicated that, and to what extent, the changes in WSS resulting from the loss as well as the subsequent regaining of blood flow during shock and resuscitation accounted for endothelial activation [7]. The abrupt reflow-related enhancement of cytokine-induced endothelial proinflammatory activation supported the view that sudden regain of flow during resuscitation had an aggravating effect on endothelial activation, which might play a significant role in vascular dysfunction and consequent organ injury [7].

Furthermore, myocardial edema initiates during the ischemic stage but abruptly expands during the first minutes of reperfusion when the gradient between the hyperosmotic extravascular fluid and the normo-osmotic blood rapidly increases [1]. Through increasing the hydrostatic pressure within the interstitial space, this edema can lead to capillary compression and aggravation of cell damage [23]. Myocardial edema is both a consequence and a mechanism of reperfusion injury through a vicious cycle.

Optimal stent deployment is an important component in determining outcomes with PCI. Since the seminal work by Colombo et al., high pressure stent balloon inflation has been the standard method of stent deployment [24]. Use of the pressure optimization protocol during stent balloon inflation *in a recent study* was associated with better long-term outcomes, particularly for target vessel revascularization [25]. However, duration of stent deflation, and particularly the velocity of balloon deflation, has not received as much attention. In the current study, the slow deflation improves coronary flow and shrinks the size of myocardial infarction, *which might be associated with decreased coronary blood flow fluctuation or WSS and might exert a favorable effect on endothelial function, myocardial edema, and microvascular obstruction*.

Although recent meta-analyses suggest a clinical benefit of postconditioning in reducing infarct size by serial cardiac enzymes, many subsequent larger trials using advanced cardiac MRI imaging have found no acute benefit of this intervention [3, 26]. Therefore, it is unclear whether these potential improvements in surrogate markers translate into beneficial clinical outcome [3, 26]. The success of postconditioning has been associated with patient-related factors (age and gender), stenting technique (direct stenting versus predilatation), aspiration thrombectomy, and the postconditioning algorithm itself (duration of the ischemia/reperfusion cycles, time between reperfusion, and ischemic conditioning). On the other hand, a previous study indicated that ischemic postconditioning did not improve LVEF within 7 days but did improve it significantly over 3 months [27, 28]. Consistent with this result, our data showed that the slow balloon deflation strategy, in spite of its favorable effects on decreasing infarct size (determined by CK and TNI release), did not lead to *a significant improvement for LVEF* during the early period following primary PCI. And myocardial stunning might mask the beneficial effects of contractile function within days to weeks after acute myocardial infarction [28]. Therefore, more long-term follow-up data might be needed.

Actually, the concept of ischemic postconditioning is much different from the concept of slow deflation during stenting in the present study, as stenting and thus slow balloon deflation is applied at a time point after reperfusion has been established. Deferred stenting, another attempt to improve the primary PCI outcome after reperfusion restored, is still controversial for the prevention of no-flow/slow-flow or MACEs [29–33]. Therefore, more effectively adjunctive therapy is needed to enhance the benefits of primary PCI.

### 4.1. Study Limitations

First, this study was limited by the relatively small population of the STEMI patient from a single center. Due to the absence of similar studies to date, further research is required to establish the effectiveness of slow balloon deflation during stent deployment, with a larger sample size. Second, the myocardial infarct size was evaluated by the peak of CK/CK-MB or TNI and AUC of CK-MB in the present study; however, cardiac MRI had not used to examine the myocardial edema or infarct size. Third, more than 70% enrolled patients have dual antiplatelet therapy with clopidogrel, and this does not correspond to the treatment recommended by the most recent guidelines for STEMI patients. The reason is that ticagrelor had not yet entered the list of China's medical insurance and was self-funded during the study period. Fourth, we speculated that the rapid balloon deflation might increase the risk of falling off of plaque fragments and microcirculation embolization in the downstream of coronary artery. *However, the downstream filter wire was not utilized in the present study*. Fifth, the abrupt opening of the complete occluded infarction-related artery (IRA) will have a greater hemodynamic effect on the downstream vessels. We speculated that the slow balloon deflation strategy during primary PCI might have favorable effects in STEMI patients with TIMI grade 0 in IRA, and patients with partial reperfusion might weaken the benefit of slow balloon deflation. Among the patients we enrolled, in spite of not all enrolled patients with IRA TIMI grade 0, a majority of IRA (166/211) are completely occluded, in which 79/105 in the rapid balloon deflation vs 87/106 in the slow deflation (*P* = 0.967). As for the predilation during primary PCI, more than 80% lesions of IRA received predilation before stent implantation. And distal flow/pressure and WSS might be changed during the predilation procedure. However, from our clinical experience, compared with stent implantation or postdilation, balloon predilation causes less incidence of slow-flow or no-reflow. And there was no significant difference with regard to the proportion of predilation procedure and cTFC value just before stenting between the two groups. *Sixth, in general, there was no statistical difference in baseline data between the two groups (*Table 1*). However, in the BNP level, the proportions of hypertension and hyperlipidemia in the rapid balloon deflation group were slightly higher than that in the slow group, which might have a slight influence on the results*. Lastly, the slow deflation did not reduce 30-day cardiovascular events, which might be associated with fewer patients enrolled and shorter duration of follow-up. Long-term follow-up is needed to assess the influence of slow balloon deflation on MACEs.

## 5. Conclusions

This randomized controlled trial demonstrated that the slow balloon deflation during stent implantation in primary PCI improved the coronary flow and decreased infarct size in patients with STEMI. This beneficial impact was likely related to decreased coronary flow volatility or shear stress.

## Figures and Tables

**Table 1:** Baseline characteristics.

	Rapid deflation, *n* = 105	Slow deflation, *n* = 106	*P*
Age (years)	62.9 ± 13.7	61.2 ± 10.9	0.322
Male (gender)	84 (80.0)	92 (86.8)	0.185
BMI (kg/m^2^)	24.7 ± 2.2	24.4 ± 2.4	0.332
SBP (mmHg)	125 ± 25	124 ± 19	0.740
DBP (kg/m^2^)	76 ± 12	75 ± 11	0.656
Oxygen saturation (%)	97.9 ± 1.9	98.3 ± 1.7	0.216
Hemoglobin (g/l)	135.7 ± 16.8	137.5 ± 16.1	0.433
BNP (pg/ml)	190 ± 229	160 ± 146	0.267
Hypertension	62 (59.0)	57 (53.8)	0.468
Hyperlipoidemia	34 (32.4)	28 (26.4)	0.544
Diabetes	20 (19.0)	21 (19.8)	0.889
Smoking	55 (52.4)	59 (55.7)	0.631
eGFR (ml/min/1.73 m^2^)	59.5 + 8.8	60.4.9 + 8.6	0.455

Data are presented as mean ± SD or number (%) of subjects. BMI: body mass index; SBP: systolic blood pressure; DBP: diastolic blood pressure; eGFR: estimated glomerular filtration rate; BNP: brain natriuretic peptide.

**Table 2:** Angiographic characteristics.

	Rapid deflation, *n* = 105	Slow deflation, *n* = 106	*P*
Infarct-related artery			
LAD	45 (42.9)	56 (52.8)	0.147
LCX	22 (21.0)	14 (13.2)	0.135
RCA	38 (36.2)	36 (34.0)	0.735
Coronary lesion			
One-vessel	65 (61.9)	62 (58.2)	0.612
Two-vessel	31 (29.5)	35 (33.0)	0.584
Three-vessel	9 (8.5)	9 (8.5)	0.983
Killip classification			
I/II/III/IV	88/16/1/0	89/15/2/0	0.833
Symptom to FMC (h)	4.6 ± 3.4	4.1 ± 3.2	0.264
D to B (min)	74 ± 35	77 ± 34	0.481

Data are presented as mean ± SD or number (%) of subjects. LAD: left anterior descending; LCX: left circumflex; RCA: right coronary artery; FMC: first medical contact; D to B: door to balloon.

**Table 3:** PCI characteristics and medications.

	Rapid deflation, *n* = 105	Slow deflation, *n* = 106	*P*
Target lesion length (mm)	24.4 ± 9.3	24.2 ± 9.5	0.876
Proximal reference (mm)	3.3 ± 0.5	3.4 ± 0.4	0.302
Distal reference (mm)	3.1 ± 0.4	3.2 ± 0.4	0.391
Number of stent implanted	1.13 ± 0.34	1.13 ± 0.34	0.979
Inflation pressure during stent implantation (atm)	16.2 ± 2.1	16.1 ± 2.0	0.570
Predilation	92 (87.6)	90 (84.9)	0.567
Postdilation	48 (45.7)	42 (39.6)	0.371
Thrombus aspiration	21 (20.0)	24 (22.6)	0.640
Tirofiban	77 (73.3)	73 (68.9)	0.474
aspirin + clopidogrel	78 (74.3)	77 (72.6)	0.787
aspirin + ticagrelor	27 (25.7)	29 (27.4)	0.787
ACEI/ARB	92 (87.6)	93 (87.7)	0.979
Statins	88 (83.8)	86 (81.1)	0.609
Beta-blockers	90 (85.7)	92 (86.8)	0.820
Oral anticoagulation	3 (2.9)	2 (1.9)	0.683

Data are presented as mean ± SD or number (%) of subjects. ACEI/ARB: angiotensin-converting enzyme inhibitor/angiotensin II receptor blocker.

**Table 4:** Results of coronary flow.

	Rapid deflation, *n* = 105	Slow deflation, *n* = 106	*P*
TIMI before PCI			
0/1/2/3	79/10/8/8	87/2/7/10	0.111
CTFC before PCI (frames)	88 ± 23	89 ± 24	0.867
CTFC before stenting (frames)	43 ± 18	44 ± 22	0.654
CTFC post1 (frames)	28 ± 9	25 ± 8	0.035
CTFC post2 (frames)	27 ± 9	24 ± 7	0.015
CK max (*μ*/l)	4098 ± 3410	2847 ± 2204	0.004
CK-MB max (*μ*/l)	357 ± 236	272 ± 212	0.007
TNI max (ng/ml)	65.3 ± 30.7	52.4 ± 33.6	0.004
No-reflow	8 (7.6)	3 (2.8)	0.118

Data are presented as mean ± SD or number (%) of subjects. TIMI: thrombolysis in myocardial infarction; CTFC: corrected TIMI frame count; PCI: percutaneous coronary intervention; CTFC post1: CTFC immediately after stent deployment: CTFC post2: CTFC at the end of PCI; CK: creatine kinase, TNI: troponin.

**Table 5:** MACEs at 30-day.

	Rapid deflation, *n* = 105	Slow deflation, *n* = 106	*P*
Mortality	3 (2.9)	2 (1.9)	0.683
Heart failure	6 (5.7)	7 (6.6)	0.788
Myocardial infarction	1 (1.0)	0	0.498
Target vessel/lesion revascularization	1 (1.0)	1 (0.9)	1.000
Rehospitalization	10 (9.5)	9 (8.5)	0.793

Data are presented as number (%) of subjects.

## Data Availability

The data that support the findings of this study are available on request from the corresponding author.
